# Replicative Capacity of MERS Coronavirus in Livestock Cell Lines

**DOI:** 10.3201/eid2002.131182

**Published:** 2014-02

**Authors:** Isabella Eckerle, Victor M. Corman, Marcel A. Müller, Matthias Lenk, Rainer G. Ulrich, Christian Drosten

**Affiliations:** University of Bonn Medical Centre, Bonn, Germany (I. Eckerle, V.M. Corman, M.A. Müller, C. Drosten);; Friedrich-Loeffler-Institut, Greifswald-Insel Riems, Germany (M. Lenk, R.G. Ulrich)

**Keywords:** Middle East respiratory syndrome, MERS, coronavirus, livestock, intermediate host, zoonosis, zoonotic, zoonoses, viruses, cell culture, goats, camels, Arabian Peninsula

## Abstract

Replicative capacity of Middle East respiratory syndrome coronavirus (MERS-CoV) was assessed in cell lines derived from livestock and peridomestic small mammals on the Arabian Peninsula. Only cell lines originating from goats and camels showed efficient replication of MERS-CoV. These results provide direction in the search for the intermediate host of MERS-CoV.

Coronaviruses (CoV) in the genera *Alphacoronavirus* and *Betacoronavirus* (order *Nidovirales*, family *Coronaviridae*, subfamily *Coronavirinae*) infect a broad range of mammals, including humans (*1*). The human CoVs (HCoVs) HCoV-HKU1, HCoV-229E, HCoV-NL63, and HCoV-OC43 typically cause mild to moderate respiratory tract infection; however, the disease course can be more severe in a minority of patients. In 2002–2003, an epidemic of severe lower respiratory tract infection with a case-fatality rate of ≈10% was caused by severe acute respiratory syndrome (SARS)–CoV (*2*). In 2012, another CoV associated with severe respiratory disease emerged on the Arabian Peninsula and was termed Middle East respiratory syndrome (MERS)–CoV (*3*).

Both SARS-CoV and MERS-CoV are zoonotic viruses, and their presumed origin is in bats. SARS-related CoVs were identified in *Rhinolophus* spp. bats in China and Europe (*4*,*5*), and MERS-related CoVs were found in *Pipistrellus* bats in Europe and in *Neoromicia* bats in South Africa (*6*,*7*). As with SARS-CoV, it is expected that MERS-CoV might be transmitted to humans by an intermediate animal host, and neutralizing antibodies against MERS-CoV have been found in Arabian camels originating from Oman, Spain, and Egypt (*8*,*9*).

We investigated replication of MERS-CoV in cell lines of the most abundant mammalian livestock species and representative peridomestic small mammals on the Arabian Peninsula. To estimate MERS-CoV permissiveness of cell cultures derived from these animals, we compared MERS-CoV replication and infectious virus production with that in bat- and primate-derived cells known to be permissive for MERS-CoV. The MERS-CoV receptor dipeptidyl peptidase 4 (DPP-4) is expressed in epithelial cells of the lung and kidney, and patients with MERS-CoV consistently show severe involvement of both organs; thus, we focused on lung and kidney cells in potential animal hosts (*10*,*11*).

## The Study

**Figure 1:**
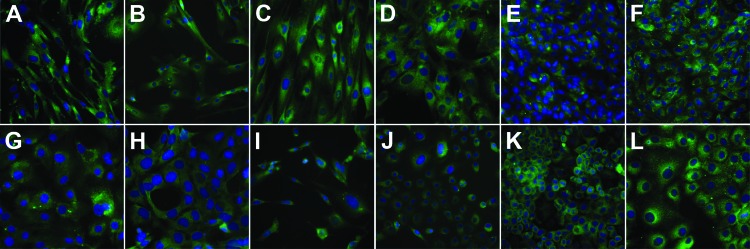
Immunofluorescence staining of the Middle East respiratory syndrome coronavirus receptor dipeptidyl peptidase 4 (DPP-4; rabbit anti-CD26/DPP-4 polyclonal antibody; Bioss Inc., Woburn, MA, USA) in cell lines used to guide the search for the intermediate host of the virus. Counterstaining of nuclei was done by using 4′,6-diamidino-2-phenylindole. Cell lines: A) ZN-R, B) ZLu-R, C) LGK-1-R, D) TT-R.B, E) PO, F) KN-R, G) KLu-R, H) MyglaAEC.B, I) Crocsu-Lu, J) PipNi, K) A549, and L) Vero E6. Magnification 400-fold.

**Figure 2:**
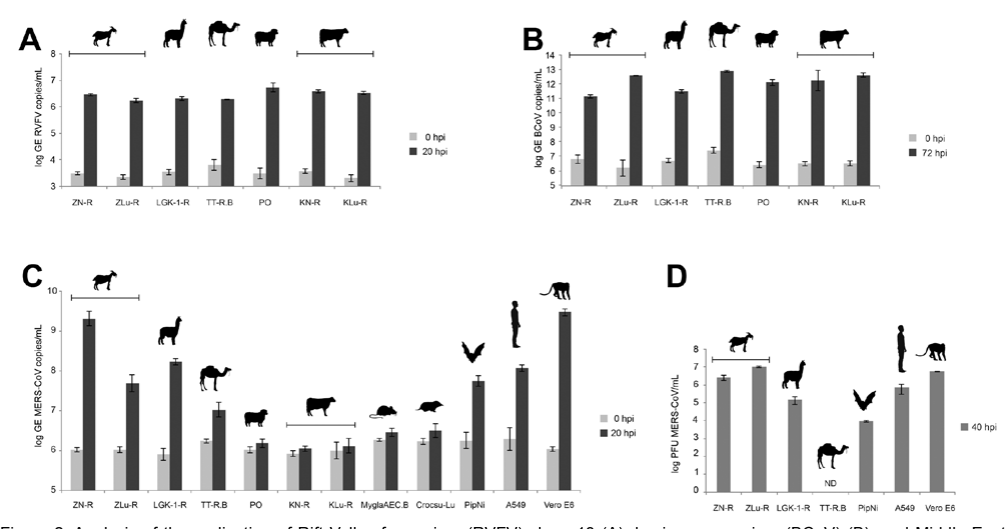
Analysis of the replication of Rift Valley fever virus (RVFV) clone 13 (A), bovine coronavirus (BCoV) (B), and Middle East respiratory syndrome coronavirus (MERS-CoV) (C) and of the production of infectious MERS-CoV particles (D) in cell lines derived from livestock and peridomestic small mammals on the Arabian Peninsula. Cell lines of human, bat, and primate origin were used as controls. Replication levels for each virus used are given as log of the genome equivalents (GEs) (A–C) or as plaque-forming units (PFUs). Vertical bars indicate ranges; horizontal bars indicate cell line origins. Using panel C as a reference, symbols represent (left to right) goat, alpaca, Arabian camel, sheep, cattle, bank vole, shrew, bat, human, and African green monkey. ND, not detected; hpi, hours postincubation.

Using enhanced respiratory personal protection equipment in a Biosafety Level 3 facility, we cultivated, in parallel, cell lines from goats, sheep, cattle, camelids (dromedary and alpaca), rodents, insectivores, bats, and human and nonhuman primates (Table). Cells were checked to ensure the absence of mycoplasma contamination and genotyped for their species of origin by sequencing of the mitochondrial cytochrome c subunit oxidase I gene (*12*). All cells expressed DPP-4, as determined by immunofluorescence staining (Figure 1) and Western blot analysis (data not shown). Because several of the ungulate cell lines had not previously been used for viral infection experiments, we determined permissiveness of all cell lines for Rift Valley fever virus (RVFV) clone 13, a virus mutant known to be attenuated yet broadly infectious for ungulate cell lines (*13*). Triplicate infections with multiplicities of infection (MOIs) of 0.5 infectious units/cell resulted in highly consistent levels between cells (maximal variation 3.2-fold) (Figure 2, panel A). In addition, to demonstrate the ability of the cells to support CoV replication, we infected all cell lines in triplicate (MOIs of 0.5) with bovine CoV strain Nebraska. The strain replicated to high levels in all cell lines; replication varied by <52.9-fold, which constitutes small relative variations in light of the overall levels of replication (Figure 2, panel B). We conducted MERS-CoV infections under the same conditions and with MOIs of 0.5. 

**Table:** Characteristics of mammalian cell lines used in a study designed to narrow the search for the intermediate mammalian host of Middle East respiratory syndrome coronavirus

Designation	Species of origin	Mammalian order	Organ of origin	Cell type
ZN-R	Goat (*Capra hircus*)*	*Artiodactyla*	Kidney	Primary
ZLu-R	Goat (*C. hircus*)*	*Artiodactyla*	Lung	Primary
LGK-1-R	Alpaca (*Llama pacos*)*	*Artiodactyla*	Kidney	Primary
TT-R.B	Arabian camel (*Camelus dromedarius*)*†	*Artiodactyla*	Umbilical cord	Immortalized
PO	Sheep (*Ovis aries*)*	*Artiodactyla*	Kidney	Immortalized
KN-R	Cattle (*Bos taurus*)*	*Artiodactyla*	Kidney	Primary
KLu-R	Cattle (*B. taurus*)*	*Artiodactyla*	Lung	Primary
MyglaAEC.B	Bank vole (*Myodes glareolus*)*	*Rodentia*	Trachea	Immortalized
Crocsu-Lu	Lesser white-toothed shrew (*Crocidura suaveolens*)*	*Soricomorpha*	Lung	Immortalized
PipNi	Common pipistrelle (*Pipistrellus pipistrellus*)*	*Chiroptera*	Kidney	Immortalized
A549	Human (*Homo sapiens*)	*Primates*	Lung	Immortalized
Vero E6	African green monkey (*Chlorocebus aethiops*)	*Primates*	Kidney	Immortalized

*Species of origin was confirmed by sequencing of the cytochrome c subunit oxidase I gene.  †Parents and the calf phenotypically resembled *C. bactrianus*. The most likely explanation is cross-breeding between the 2 species with mitochondria stemming from a *C. dromedarius* mother in one of the former generations.

In addition to livestock cell lines, we used rodent, insectivore, bat, and primate cell lines in the experiments (Table). Bat and primate cells known to be permissive for MERS-CoV served as controls. MERS-CoV–inoculated cells were incubated for 1 h and then washed twice before supernatant was harvested (0 h after incubation) (Figure 2, panel C). We quantified virus replication by using the *upE* assay (*14*), a MERS-CoV real-time reverse transcription PCR that screens upstream of the *E* gene. Replication was seen in lung and kidney cell lines derived from goats (*Capra hircus*) and in umbilical cord and kidney cells from camelids (*Camelus dromedarius* and *Llama pacos*) (Figure 2, panel C). Efficient replication (>9.3 log_10_ virus RNA genome equivalents/mL of cell culture supernatant) was seen in the goat kidney cells; this replication level was similar to that in Vero E6 cells (interferon-deficient primate kidney cells). Goat lung cells, alpaca kidney cells, and dromedary umbilical cord cells also showed strong replication, but virus did not replicate in sheep, cattle, rodent, or insectivore cells. In addition, expression of the receptor, DPP-4, was confirmed in all cells, including the nonpermissive sheep, cattle, rodent, and insectivore cells. 

After following virus growth in all permissive cells for another 20 h, we harvested supernatants and, to confirm the production of infectious virus particles, we titrated the supernatants by using a plaque assay in Vero cells. MERS-CoV replication was seen in all permissive cells except TT-R.B (Figure 2, panel D), and all permissive cells showed cytopathic effects. The highest production of virus particles was in goat lung and kidney cells (1.0 × 10^7^ and 2.7 × 10^6^ PFU/mL, respectively). This level of replication was comparable to that in human lung cells (A549) and Vero E6 (Figure 2, panel D). 

## Conclusions

Transmission of MERS-CoV between humans is still limited, and the identification of an intermediate animal host could enable the development of public health measures to prevent future spread of the virus among humans. Although MERS-CoV neutralizing antibodies have been detected in camels from Oman, Spain, and Egypt, the virus has not previously been detected in camels (*8**,**9*). An informed focusing of investigations on a select group of species, such as camels, could benefit epidemiologic investigations. To identify potential intermediate host species of MERS-CoV, we used in vitro testing to determine virus permissiveness in select cell culture models. In general, cell lines cannot depict the full pathogenicity of in vivo infection because infection is influenced by epithelium-specific differentiation of target cells and the presence of immune cells. However, for viruses such as CoVs, whose tropism is believed to be determined mainly by the availability of an appropriate entry receptor (*10*), epithelial cell cultures could indeed constitute valid surrogates of virus permissiveness in vivo. With these limitations in mind, our results are in concordance with the findings of MERS-CoV neutralizing antibodies in camels and with information regarding patient contact with animals in reports of 2 human cases of MERS-CoV infection (*11*,*15*). One of the patients owned a farm on which camels and goats were kept. Before onset of his own illness, the patient reported illness in several goats on his farm. The patient did not have direct contact with animals, but he reported having eaten goat meat and having had contact with one of the animal caretakers, who suffered from respiratory disease (*15*). The second patient reported direct contact with a diseased camel shortly before onset of his symptoms (*11*).

In our study, production of infectious virus particles was seen in goat lung and kidney cells and in camelid kidney cells. Excretion patterns indicative of kidney infection should be investigated once further clues to the identity of the MERS-CoV animal reservoir become available. Our preliminary findings suggest that ungulates, such as goats and camels, are a possible intermediate host of MERS-CoV; thus, exposure to urine and feces from these animals might constitute a source of human infection. Moreover, food products derived from these animals (e.g., meat and milk) should be tested for their potential to transmit MERS-CoV. The results of our study suggest that investigations into the MERS-CoV animal reservoir and intermediate host should focus on caprid (e.g., goats) and camelid hosts, and we identified several new cell lines for use in virus isolation studies.

## References

[1] Woo PC, Lau SK, Huang Y, Yuen KY. Coronavirus diversity, phylogeny and interspecies jumping. Exp Biol Med (Maywood). 2009;234:1117–27. 10.3181/0903-MR-94 19546349

[2] Drosten C, Gunther S, Preiser W, van der Werf S, Brodt HR, Becker S, Identification of a novel coronavirus in patients with severe acute respiratory syndrome. N Engl J Med. 2003;348:1967–76. 10.1056/NEJMoa030747 12690091

[3] de Groot RJ, Baker SC, Baric RS, Brown CS, Drosten C, Enjuanes L, Middle East respiratory syndrome coronavirus (MERS-CoV): announcement of the Coronavirus Study Group. J Virol. 2013;87:7790–2. 10.1128/JVI.01244-13 23678167 PMC3700179

[4] Lau SK, Woo PC, Li KS, Huang Y, Tsoi HW, Wong BH, Severe acute respiratory syndrome coronavirus-like virus in Chinese horseshoe bats. Proc Natl Acad Sci U S A. 2005;102:14040–5. 10.1073/pnas.0506735102 16169905 PMC1236580

[5] Li W, Shi Z, Yu M, Ren W, Smith C, Epstein JH, Bats are natural reservoirs of SARS-like coronaviruses. Science. 2005;310:676–9. 10.1126/science.1118391 16195424

[6] Annan A, Baldwin HJ, Corman VM, Klose SM, Owusu M, Nkrumah EE, Human betacoronavirus 2c EMC/2012-related viruses in bats, Ghana and Europe. Emerg Infect Dis. 2013;19:456–9. 10.3201/eid1903.121503 23622767 PMC3647674

[7] Ithete NL, Stoffberg S, Corman VM, Cottontail VM, Richards LR, Schoeman MC, Close relative of human Middle East respiratory syndrome coronavirus in bat, South Africa. Emerg Infect Dis. 2013;19:1697–9. 10.3201/eid1910.130946 24050621 PMC3810765

[8] Perera RA, Wang P, Gomaa MR, El-Shesheny R, Kandeil A, Bagato O, Seroepidemiology for MERS coronavirus using microneutralisation and pseudoparticle virus neutralisation assays reveal a high prevalence of antibody in dromedary camels in Egypt. Euro Surveill. 2013;18:20574 . 24079378 10.2807/1560-7917.es2013.18.36.20574

[9] Reusken CB, Haagmans BL, Muller MA, Gutierrez C, Godeke GJ, Meyer B, Middle East respiratory syndrome coronavirus neutralising serum antibodies in dromedary camels: a comparative serological study. Lancet Infect Dis. 2013;13:859–66. 10.1016/S1473-3099(13)70164-6 23933067 PMC7106530

[10] Raj VS, Mou H, Smits SL, Dekkers DH, Muller MA, Dijkman R, Dipeptidyl peptidase 4 is a functional receptor for the emerging human coronavirus-EMC. Nature. 2013;495:251–4. 10.1038/nature12005 23486063 PMC7095326

[11] Drosten C, Seilmaier M, Corman VM, Hartmann W, Scheible G, Sack S, Clinical features and virological analysis of a case of Middle East respiratory syndrome coronavirus infection. Lancet Infect Dis. 2013;13:745–51. 10.1016/S1473-3099(13)70154-3 23782859 PMC7164791

[12] Alcaide M, Rico C, Ruiz S, Soriguer R, Munoz J, Figuerola J. Disentangling vector-borne transmission networks: a universal DNA barcoding method to identify vertebrate hosts from arthropod bloodmeals. PLoS ONE. 2009;4:e7092 . 10.1371/journal.pone.0007092 19768113 PMC2740869

[13] Muller R, Saluzzo JF, Lopez N, Dreier T, Turell M, Smith J, Characterization of clone 13, a naturally attenuated avirulent isolate of Rift Valley fever virus, which is altered in the small segment. Am J Trop Med Hyg. 1995;53:405–11 . 7485695 10.4269/ajtmh.1995.53.405

[14] Corman VM, Eckerle I, Bleicker T, Zaki A, Landt O, Eschbach-Bludau M, Detection of a novel human coronavirus by real-time reverse-transcription polymerase chain reaction. Euro Surveill. 2012;17:20285 . 23041020 10.2807/ese.17.39.20285-en

[15] Buchholz U, Muller MA, Nitsche A, Sanewski A, Wevering N, Bauer-Balci T, Contact investigation of a case of human novel coronavirus infection treated in a German hospital, October–November 2012. Euro Surveill. 2013;18:20406 . 23449231

